# Evaluation of urodynamic parameters after sling surgery in men with post-prostatectomy urinary incontinence

**DOI:** 10.1590/S1677-5538.IBJU.2017.0243

**Published:** 2018

**Authors:** Odair Gomes Paiva, João Paulo Cunha Lima, Carlos Alberto Bezerra

**Affiliations:** 1 Disciplina de Urologia, Faculdade de Medicina do ABC, Santo André, SP, Brasil

**Keywords:** Suburethral Slings, Surgical Procedures, Operative, Urinary Incontinence

## Abstract

**Objective:**

To evaluate urodynamic alterations after sub-urethral sling surgery (SSU) in patients with post-prostatectomy urinary incontinence (PPUI).

**Materials and Methods:**

We evaluated data of 22 patients submitted to radical prosta- tectomy (RP) or transurethral resection of prostate (TURP) that presented post-surgical urinary incontinence and were treated with SSU implant in a pilot study previously performed in our institution.

**Methods:**

Those patients with PPUI were evaluated by urodynamic exam (UD) before and after surgery, and the parameters were compared, including uroflow, cystometry and micturition study. Exclusion criteria included patients without pre-operatory urodynamic study, those with urethral stenosis, those not healed of prostate cancer, pa- tients without clinical conditions to be submitted to urodynamic study and those with severe neurological diseases or that refused to sign the consent form. Results were analyzed statistically by Fisher, Wilcoxon or Mann-Whitney tests.

**Results:**

During free uroflow, none parameters showed any statistical significant differ- ences. During cystometry, there were also no statistical differences and the same was observed at pressure versus flow study; the exception was at maximal flow detrusor pressure (PdetQmax), that was lower at post-operatory (p=0.028). In relation to the presence of urinary dysfunctions associated to PPUI, we observed a significant reduc- tion of detrusor overactivity (p=0.035) in relation to pre-operatory period.

**Conclusion:**

SSU surgery significantly reduced detrusor overactivity and PdetQMax; however, there were no alterations of other evaluated urodynamic parameters.

## INTRODUCTION

Post-prostatectomy urinary incontinence (PPUI) is a common complication of surgical treatment of patients with prostate cancer or benign prostatic hyperplasia and cause a negative impact on quality of life (1).

PPUI is defined by International Continence Society (ICS) as an involuntary loss of urine during strain following prostatic surgery, that can be single or associated to other vesical dysfunctions (2–4).

The main and more prevalent cause of PPUI is radical prostatectomy (RP), and its incidence varies from 2.5% to 67% (5, 6).

Some studies show that the main urethral and vesical dysfunctions that occur after RP include: intrinsic sphincter deficiency, detrusor overactivity, loss of complacency and loss of detrusor contractility (7, 8).

PPUI treatment includes conservative measures (physiotherapy, for example), sub-urethral sling surgeries (SSU) and artificial urinary sphincter (AUS). Conservative treatment speeds recovery of slight PPUI in the first months following surgery, and there is indication of surgical treatment for patients without improvement (9). The most efficient surgical treatment is AUS implant, but with higher costs. Also, some patients decide for the use of a mechanical device (10). In that con-text, SSU surgeries were proposed and nowadays they include two types, according to their mechanism of action: those adjustable and compressive, and those functional or non-compressive (11).

There are many controversial and uncertain aspects regarding those action mechanisms of SSU; they have been studied by urodynamic exams and also by magnetic nuclear resonance of pelvis, to evaluate position and length at membranous urethra (12). In relation to urodynamic evaluation, there are few reports of the whole exam, and the authors choose to report only some urodynamic data. Therefore, new studies are justified for better evaluation of SSU effects (13).

We analyzed patients submitted to SSU by urodynamic studies, to improve understanding of the effects of surgery in the main parameters of the exam, trying to identify potential prognostic factors or action mechanisms.

## OBJECTIVE

To evaluate urodynamic alterations caused by SSU surgery in patients with post-prostatectomy urinary incontinence.

## MATERIALS AND METHODS

This is an observational and retrospective study of 22 patients previously submitted to SSU in our institution. From December 2010 to June 2013, all patients with PPUI submitted to SSU sur-gery were invited to participate. Age varied from 50 to 74 years (median 66 years); all patients were submitted to urodynamic study before and after surgery. Pre-operatory exam was performed after at least 8 months of history of PPUI and at least 6 months after SSU surgery. Patients were part of another pilot study that compared two SSU techniques: compressive and adjustable versus non-compressive (1). In summary, patients with any intensity of PPUI were randomized and prospectively treated with SSU. In that study, there were no significant statistical differences of objective criteria (pad test, use of sanitary napkins) but Advance® sling had better subjective results (satisfaction).

Inclusion criteria: patients that signed the Free Consent Form, patients no irradiated, without urethral stenosis and/or previous stenosis but surgically treated by SSU, those still with PPUI or healed by SSU surgery. Exclusion criteria: patients without pre-operatory urodynamic study, those with urethral stenosis not treated, with active prostate neoplasia, without clinical conditions for the procedure or with severe neurological diseases.

For this study, PPUI was defined as any urinary loss confirmed by urodynamic study and/or pad test that promoted the patient to desire treatment.

The following slings were used: Argus T® - Promedon – Argentina (compressive and adjustable) or Advance® - A M S - USA (non-compressive). They were available when our Public Institution concluded bidding process.

All patients were submitted to urodynamic study, that included three phases: uroflow (maximum flow (QMax), post-micturition residual urine, urinary volume), cystometry (maximum cystometric capacity (MCC), first micturition desire, volume at normal micturition desire, urinary loss pressure (VLPP) and flow/pressure study (detrusor pressure at maximum flow (PdetQmax), maximum flow (Qmax), urinary volume and post-micturition residual urine). The following equipments were used: Alacer- Uranus II and Dynamed- Dynapack MPX816 urodynamic devices, that were managed according to the recommendations of the International Continence Society (ICS) (Schafer (14, 15)).

The first phase of the study was free uro-flow: when the patient had a moderate desire to urinate, he was oriented to urinate freely in the equipment and, following micturition, the computer program provided a graphic for analysis.

In the second phase of the exam, cystometry, the patient was positioned in a bed in supine position, for genital region asepsis. Five to 10 mL of lidocaine gel was introduced in the urethra and two urethral catheters were positioned (6 and 8Fr) and fixed at the penis, and the urinary residue was measured. Next, a rectal catheter was introduced for measurement of abdominal pressure. Lastly, the patient was seated in an appropriated chair and the catheters were attached to the equipment and the cystometry was initiated.

Sterile distilled water was introduced in the 8Fr urethral catheter and the filling was monitored; it was asked to the patient inform the first micturition desire, the normal desire and the maximum desire. When the last was reached, he was asked to cough (strain maneuver) to evaluate the presence of urinary loss. If there were no losses, the 8Fr catheter was removed and the stress maneuver was again performed to verify any urinary loss.

Next, the patient was asked to urinate for the flow/pressure study; after that, residual urine was measured and the catheters were removed, ending the exam.

Following the urodynamic study, the patients received antibiotic prophylaxis with norfloxacin 400 mg every 12 hours for three days, and were clinically followed up to identify possible signs of secondary infection.

The results were statistically analyzed by the Fisher, Wilcoxon and Mann-Whitney tests, with a significant level of 5%.

The study protocol was approved by the Ethical Research Committee of the Institution.

## RESULTS

All 22 patients with PPUI submitted to SSU surgery were included in the present study. Table-1 shows median age, type of prostate surgery and median time from prostatectomy to sling surgery.

**Table 1:** Time after prostate surgery, age and type of SSU surgery.

	Argus T®	Advance®
Age (medium)	62.55 (52 to 74 years)	62.09 (50 to 71 years)
Time after prostate surgery (medium)	53.82 (8 to 98 months)	52.18 (12 to 187 months)
Prostate Surgery	10 RP / 1 TURP	11 RP

**RP** = radical prostatectomy; **TURP** = transurethral ressection of prostate

Two patients died due to acute myocardial infarction; one after six months of the procedure and the other after 12 months, both without any relation to SSU surgery.

After at least six months of surgery for PPUI correction, 4 of the 22 patients with pre-operatory UD were excluded, since 2 died and 2 developed urethral stenosis. Of those, 3 had been submitted to Argus T SSU® and one to Advance® implant.

Table-2 shows the urodynamic parameters during free uroflow, cystometry and flow/pressure study; the only parameter with significant difference was detrusor pressure at maximal flow (PDetQ-max) (p<0.028).

**Table 2:** Bladder dysfunction: comparison of pre and post-operatory.

	Pre	Post	p* (pre x post)
Complacence Deficit	4(18%)	2(10.5%)	0.180
Detrusor overactivity	10(45.4%)	2 (10.5%)	0.035*
Lowered maximum cystometric capacity	6(27.2%)	4 (21%)	0.414
Hypocontractility	2(9%)	6 (31.5%)	0.180
Obstruction	3 (13.6%)	3 (15.7%)	1.000

**p** - descriptive level of probability at non-parametric Wilcoxon test.

In relation to the presence of urinary dysfunction, it was observed a reduction of detrusor overactivity from 45.4% to 10.5% of patients (Table-3).

**Table 3:** Comparison of pre and post-operatory urodynamic results.

Variable		n	Medium	sd	Minimum	Maximum	P25	Median	P75	p(<0.05) *
**Qmax (free flow) (mL)**										
	Pre	19	11.12	13.76	4.00	55.20	0.00	5.90	19.00	0.877
	Post	19	11.58	8.88	0.00	32.70	4.70	11.00	17.60	
**Post micturition urinary residue (free flow) (mL)**										
	Pre	19	8.16	20.90	0.00	90.00	0.00	0.00	10.00	0.503
	Post	19	13.68	27.28	0.00	100.00	0.00	0.00	20.00	
**VLPP (cmH_2_O)**										
	Pre	18	51.50	38.56	0.00	140.00	15.70	50.00	78.75	0.356
	Post	18	74.00	73.64	0.00	200.00	0.00	62.00	155.50	
**Maximum cystometric capacity (mL)**										
	Pre	18	333.44	77.01	158.00	450.00	298.75	335.50	400.00	0.052
	Post	18	289.44	91.30	123.00	403.00	202.25	300.00	391.25	
**Qmax (f X P) (mL/s)**										
	Pre	18	17.12	12.19	3.00	44.00	8.60	13.00	24.65	0.067
	Post	18	10.34	6.69	0.00	23.30	4.98	9.65	15.35	
**Residual urine (Fxp)( mL)**										
	Pre	18	51.11	88.99	0.00	300.00	0.00	5.00	58.75	0.507
	Post	18	33.50	72.27	0.00	300.00	0.00	4.00	40.50	
**P. Det. Qmax(cmH_2_O)**										
	Pre	18	27.76	14.70	11.00	55.00	15.50	24.90	36.95	0.028*
	Post	18	15.10	12.20	0.00	38.00	3.50	15.90	25.85	
**Urinary volume at free flow (mL)**										
	Pre	17	153.32	190.92	22.20	657.50	35.10	75.00	238.50	0.102
	Post	17	206.31	175.34	42.20	699.00	62.00	221.00	312.60	
**Bladder volume at normal desire (mL)**										
	Pre	19	245.37	85.42	13.00	365.00	200.00	278.00	300.00	0.344
	Post	19	216.53	85.24	0.00	390.00	167.00	220.00	261.00	
**Urinated volume at flow/ pressure study (mL)**										
	Pre	16	338.46	89.56	132.00	388.00	275.75	317.50	379.63	0.173
	Post	16	242.38	98.44	123.00	490.30	194.15	286.90	317.23	

**Qmax** = maximum flow; **VLPP** = urinary loss pressure; **n** = number of sample; **sd** = standard deviation; **P25** = value that is preceded by 25% of values; **P75** = value that is preceded by 75% of values; **p** = descriptive level of probability at non-parametric Wilcoxon test.

After surgical procedures, the following results were observed: among patients submitted to SSU Advance® - Qmax varied from 2.6 to 30 cmH_2_O and the urinary residue varied from 0 to 42 mL. In relation to SSU Argus T, Qmax varied from 0 to 23.3 mL/s and Pdet/Qmax from 0 to 38 cm H_2_O, and the urinary residue varied from 0 to 300 mL.

When SSU pre and post-surgical results were singly compared, differences were not significant, except Pdet/Qmax of Advance® patients, that presented a statistical significant reduction.

When the results of both SSU types were compared (Argus T® x Advance®) it was not also observed any statistical difference.

## DISCUSSION

Post-prostatectomy urinary incontinence (PPUI) is a complication of prostatic surgeries (RP or TURP). Many etiologic factors are involved, but they are not well elucidated (14).

Literature shows that the main cause of PPUI is sphincter deficiency, but that may be associated to any kind of urinary dysfunction (for example, detrusor overactivity) (2). Méndez Rubio et al. (16) suggested that these findings probably reflect a secondary urinary dysfunction caused by bladder denervation during surgery. Stavropoulos et al. (17) considered age as an important factor, since it causes histological changes of bladder muscle and connective tissue. The alterations, associated to those produced by radical prostatectomy cause changes in sphincter function that can be identified by some urodynamic parameters (sphincter deficiency, detrusor overactivity, hypocontractility, etc) and can cause incontinence. Median age of those studied patients was 62 years, and being not that old, we believe that this is not such a so prevalent factor for PPUI.

Urinary dysfunction may be associated or not to sphincter deficiency, as observed by Jura et al. (10) in 15% of patients with PPUI. Barniou et al. (18) observed that urinary dysfunction may be observed even after prostatic surgeries, in a study where they reported persistence of hypocontractility in 25% and complacence deficit in 28.1% after 3 years. Similarly to our results, after more than 12 months of RP, it was observed a complacence deficit in 18% of patients and hypocontractility in 9% and it was not clear if those occurred due to bladder dysfunctionalization secondary to PPUI or to other factors.

In the last few years, SSU were designed and reformulated as an option of AUS 800® artificial sphincter, due to their promising results and lower costs, that consolidated them as an option for the treatment of PPUI (1, 19).

In relation to what literature shows regarding action mechanisms of SSU, it is suggested that AdVance® sling mash may modify the dynamics of bulbar urethra (repositioning and increase of rabdosphincter length) resulting in functional improvement of sphincter (16); differently, implant of SSU Argus T® would change angulation and urethral compression (20). Therefore, Advance Sling® doesn't have a compressive action, as observed by Bauer et al. (21); they evaluated the pre and post-surgical urodynamic parameters of patients treated with Advance® sling and observed that residual urine after initial free flow did not alter after treatment and postulated that this kind of sling did not produce a compressive effect. This non-obstructive effect was also proven by Ullrich and Comiter (4) and Davies et al. (22), that did not identify any significant change of maximum flow, pressure at maximum flow and post-micturition urinary residue.

Although literature assigns a compressive effect to Argus T Sling, Rehder et al. (23) showed that maximum flow during flow/pressure study did not alter significantly at post-operatory period. The authors believe that it is caused by the big angle produced by the sling, providing a limited perpendicular strength. The movement does not “strangle” the urethra and, therefore, it is less possible to cause tissue ischemia, lowering the chance of obstruction and atrophy.

The results observed in our study of patients submitted to Argus T cannot be considered signi-ficant for obstruction and also had no statistical difference. The only parameter with a significant difference (for less) was Pdet/Qmax: 27.76 cmH_2_O pre-op to 15.10 cmH20 post-op (p=0.028), opposing the hypothesis of obstruction or urethral compression. The small number of patients studied does not allow a definitive conclusion of this finding. One hypothesis is that SSU are not really compressive, since in some healed patients Pdet/Qmax did not raise; another possibility is that in more adequate samples with a larger number of patients, urodynamic alteration will be more clear. Literature is also rare in this aspect and new studies are needed to correct these limitations.

When Horstman et al. (11) consider SSU Argus T theoretically compressive, they refer to the tension provided by the SSU to increase continence produced at intra-operatory. It is suggested that this kind of SSU may be used in more severe incontinence. According to these authors, urodynamic study registers increase of maximum urethral closure pressure (what we did not measure in our present study) and flow reduction, but that does not correspond to obstruction at urodynamic evaluation. Still, as mentioned, our patients submitted to SSU Argus T® did not show any flow reduction.

Only detrusor overactivity at post-operatory urodynamic study was significant. It was probably caused by improvement of PPUI, due to resolution of sphincter deficiency, that resulted in a resolution of sphincter deficiency, extinguishing afferent urethral stimulation that would induce to involuntary reflex contraction that caused detrusor overactivity (24).

Urodynamic alterations due to SSU surgeries are not still completely clarified, and probably this is the cause of controversial aspects of the action mechanism of these surgeries. Therefore, future studies should focus on broadening of uro-dynamic parameters evaluated, with more better measures of urethral function using video-urody-namics, or measurement of urethral pressure, bigger series of patients, and finally, the association with other factors, such as length and position of sphincter unity pre and post-surgical of SSU using Magnetic Nuclear Resonance.

## CONCLUSIONS

SSU produced significant alterations of detrusor pressure during maximum flow, and in relation to micturition dysfunction, a significant reduction of detrusor overactivity.
